# Supplementary material for: GLUT1 overexpression in CAR-T cells induces metabolic reprogramming and enhances potency
Source: Nat Commun. 2024 Oct 6;15:8658. doi: 10.1038/s41467-024-52666-y (PMC11456602; doi:10.1038/s41467-024-52666-y)
Supplement: Supplementary file 1 — Supplementary Information [file 41467_2024_52666_MOESM1_ESM.pdf]

Supplementary Figure 1

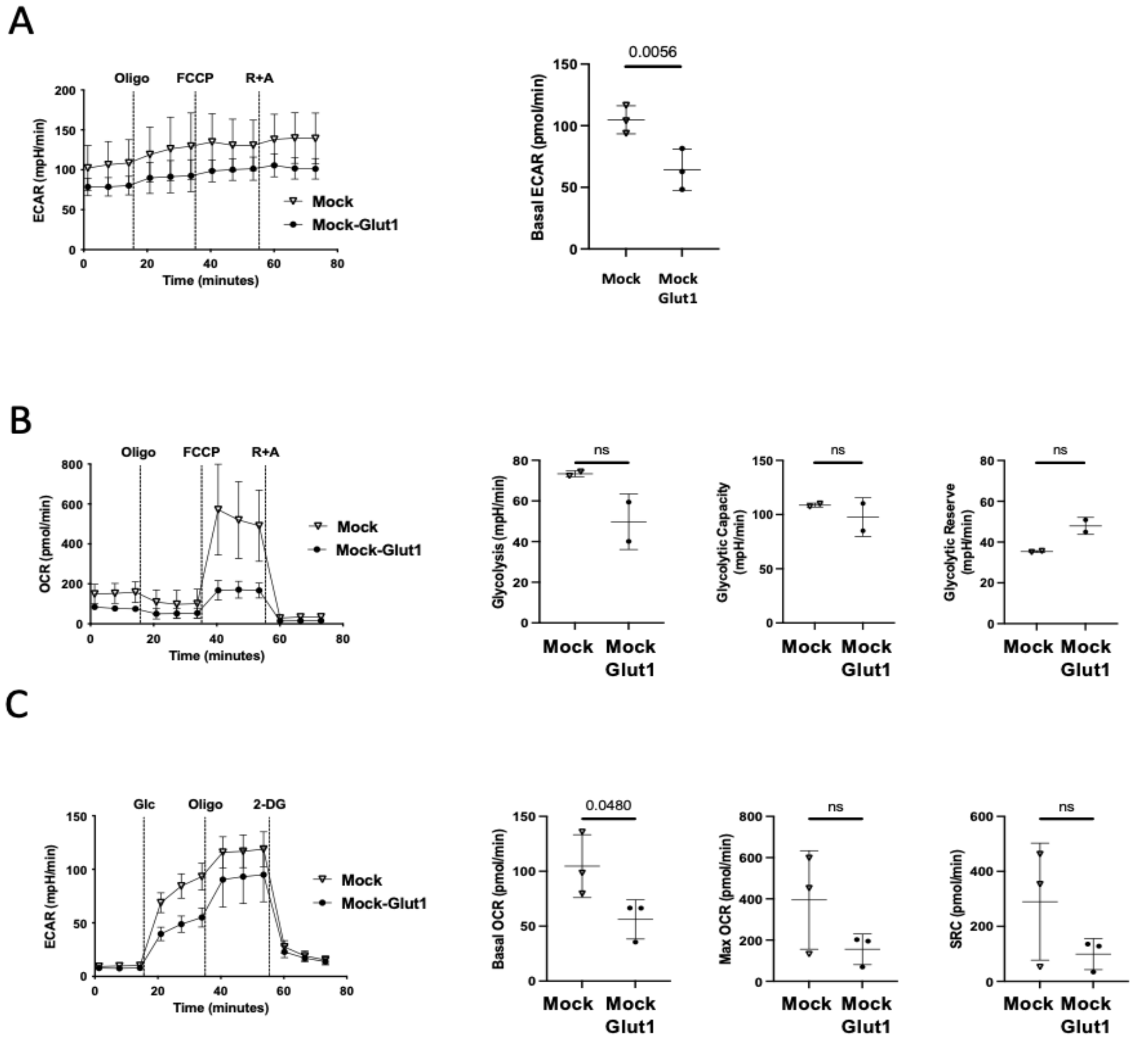

Supplemental Figure 1: GLUT1 overexpression enhances glucose uptake and oxidative phosphorylation, related to Figures 1 & 2

(A) (LEFT) Representative extracellular acidification rate (ECAR) measured using Seahorse for Mock T cells ± GLUT1OE on day 14 from one donor. (RIGHT) Pooled data for basal ECAR. Data are representative of one experiment with n = 3 donors. P values determined by unpaired two-tailed t-tests. Error bars represent SD.

(B) (LEFT) Representative ECAR (Glycolytic Stress Test) measured using Seahorse for Mock T cells ± GLUT1OE on day 12 from one donor. (RIGHT) Pooled glycolysis, glycolytic capacity and reserve data. Data are representative of one experiment with n = 2 donors. P values determined by unpaired two-tailed t-tests. Error bars represent SD.

(C) (LEFT) Representative oxygen consumption rate data measured using Seahorse for Mock T cells on day 12. (RIGHT) Quantitative data representative of one experiment with different donors; n = 3. OCR- Oxygen Consumption Rate; SRC- Spare Respiratory Capacity. P values determined by unpaired two-tailed t-tests. Error bars represent SD.

Supplementary Figure 2

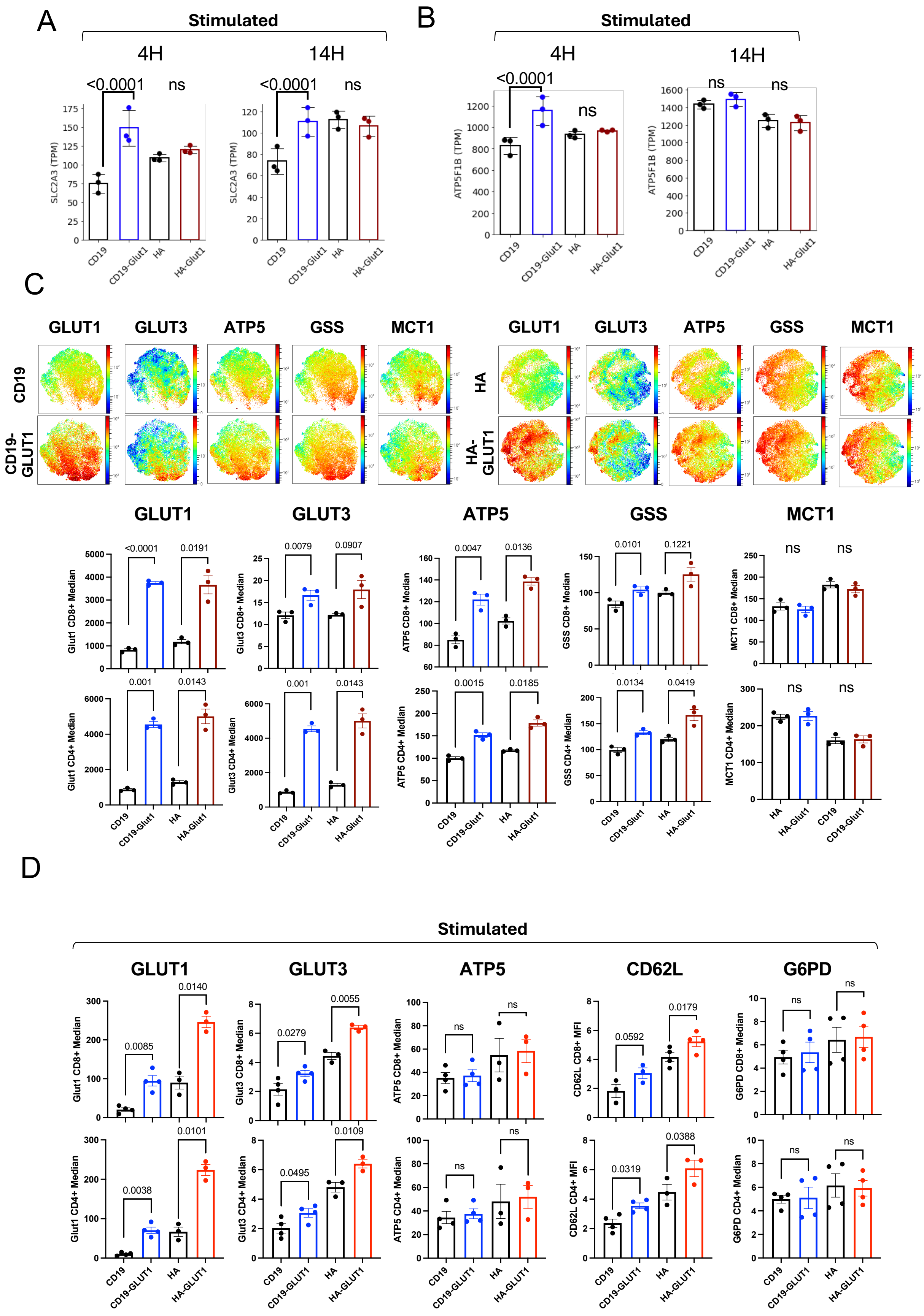

Supplemental Figure 2:

(A) TPMs of SLC2A3 (GLUT3) transcripts for CD19 and HA ± GLUT1OE CAR-T cells stimulated for (LEFT) 4 and (RIGHT) 14 hours with anti-idiotype. Data from n=3 donors. P values calculated using DESeq2.

(B) TPMs of ATP5F1B (ATP Synthase F1 subunit beta) transcripts for CD19 and HA ± GLUT1OE CAR-T cells stimulated for (LEFT) 4 and (RIGHT) 14 hours with anti-idiotype. Data from n=3 donors. P values calculated using DESeq2.

(C) (TOP) CyTOF analysis of electronically sorted CD8+ CD19 and HA ± GLUT1OE CAR-T cells at day 14. 20,000 or maximum of CD8+ HA CAR T cells from each donor (n=3) were organized by their combined expression of 12 metabolic markers using UMAPs. Donors matched and concatenated into 250,385 randomly sampled total events. (BOTTOM) Bar graphs representing median expression of indicated markers in CD8+ and CD4+ CAR-T cells. Total of n=3 donors shown. P values determined by paired t-tests. Error bars represent SEM.

(D) Mass cytometry (CYTOF) analysis of indicated metabolic proteins. Bar graphs representing median expression of indicated markers in (TOP) CD8+ (BOTTOM) CD4+ CAR-T cells stimulated for 4 hours with idiotype on day 16. Total of n=3-4 donors shown. P values determined by paired t-tests. Error bars represent SEM.

Supplementary Figure 3

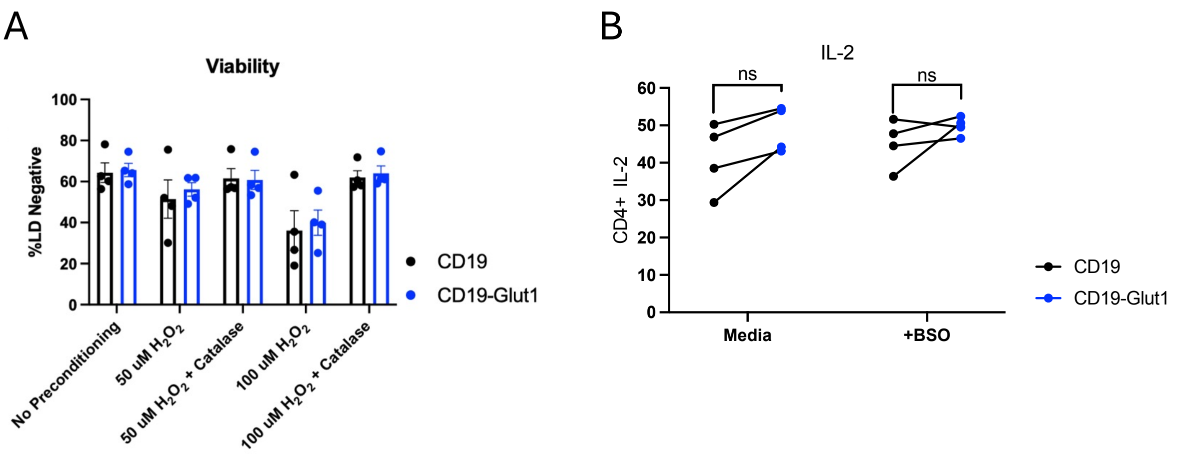

Supplemental Figure 3:

(A) Pooled data of percent live cells in response to H_2O_2 preconditioning and Nalm6 challenge.

(B) Intracellular IL-2 staining for CD4+ CD19 ± GLUT1OE CAR-T cells stimulated ± BSO. Cells were stimulated via 1 ug /mL plate bound anti-idiotype on day 14. Data from n=4 donors. P values determined by paired t-tests. Error bars represent SEM.

Supplementary Figure 4

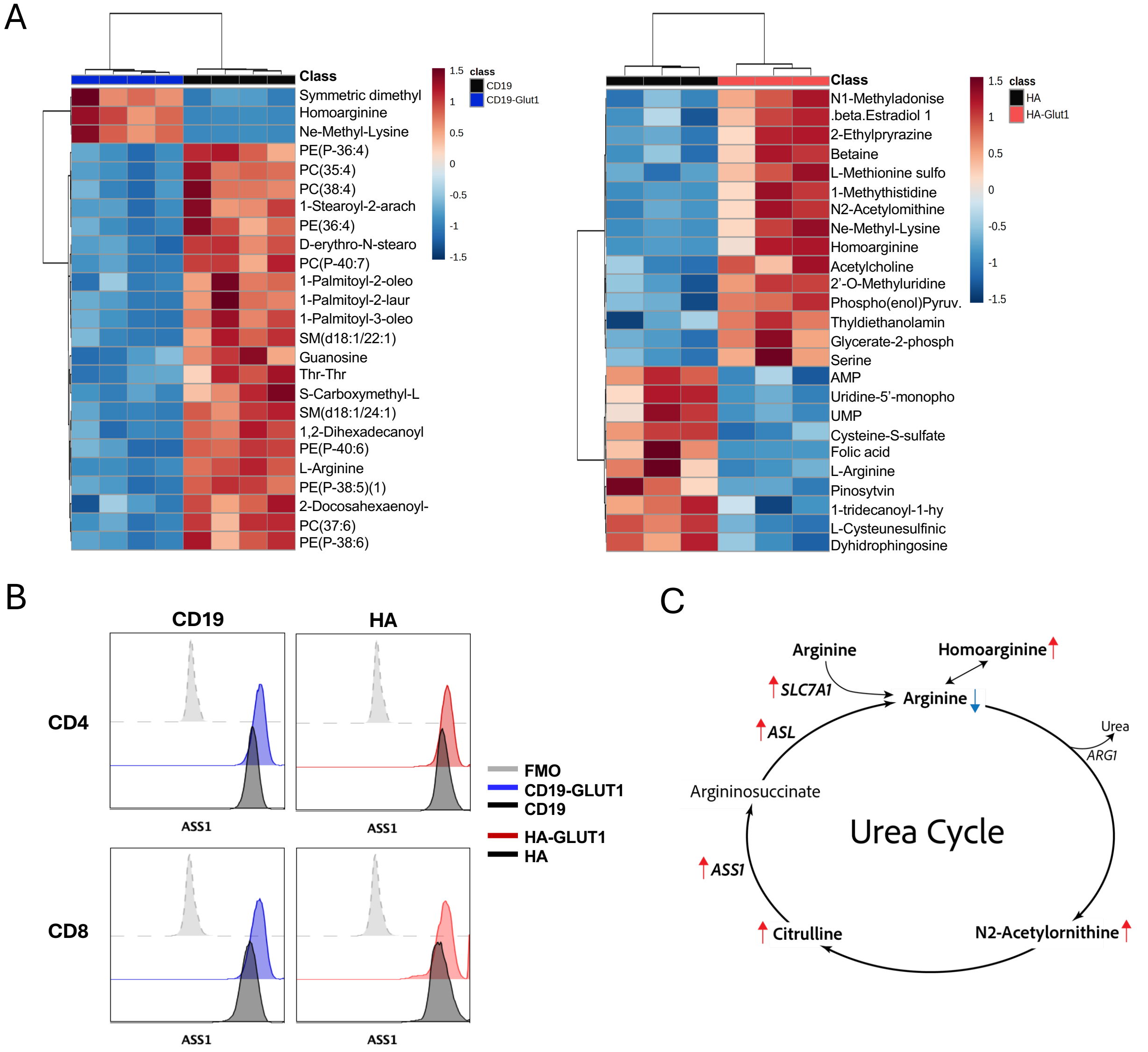

Supplemental Figure 4:

(A) Heatmap of LC-MS metabolomics data representing 25 most significantly down or upregulated metabolites in electronically sorted (LEFT) CD19 ± GLUT1OE and (RIGHT) HA ± GLUT1OE CAR-T cells on day 14.

(B) Flow cytometric analysis of intracellular expression of Argininosuccinate synthase 1 (ASS1) in CD19 and HA ± GLUT1OE CAR-T cells on day 16.

(C) Urea Cycle pathway highlighting differential findings. Red arrows indicative of enrichment. Blue arrows indicative of metabolites less abundant in GLUT1OE CAR-T cells.

Supplementary Figure 5

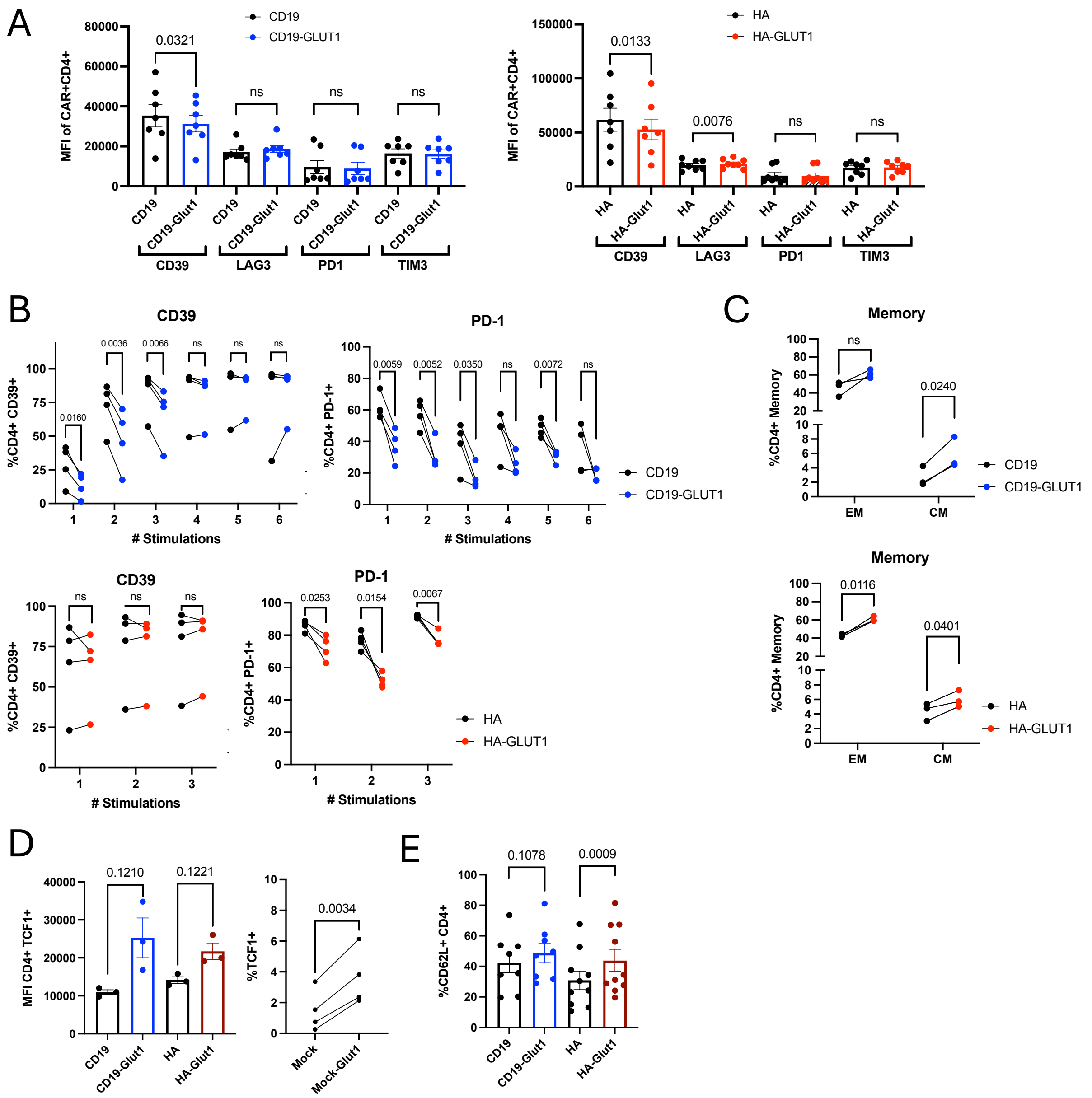

Supplemental Figure 5

(A) MFI of exhaustion markers CD39, LAG3, PD1 and TIM3 expressed by CD4+ (LEFT) CD19 or (RIGHT) HA ± GLUT1OE CAR-T cells on day 14. Pooled data of n=14 donors. P values determined by paired two-tailed t-tests. Error bars represent SEM.

(B) Flow cytometry measurements of CD39 and PD-1 of $CD4^+$ (TOP) CD19 and (BOTTOM) HA CAR-T cells ± GLUT1OE after each stimulation. P values determined by paired two-tailed t-tests. Error bars represent SEM.

(C) Memory formation data of serially rechallenged $CD4^+$ CAR-T cells after (TOP) 4 stimulations for CD19 ± GLUT1OE and (BOTTOM) 3 stimulations for HA ± GLUT1OE. EM = effector memory, CM = central memory. P values determined by paired two-tailed t-tests. Error bars represent SEM.

(D) Pooled intracellular expression of TCF1 in CD19 or HA CAR or Mock ± GLUT1OE T cells on day 16. Data from n=3-4 donors. P values determined by paired two-tailed t-tests. Error bars represent SEM.

(E) Pooled surface expression of CD4+ CD62L in CD19 or HA ± GLUT1OE CAR-T cells on day 16. P values determined by paired two-tailed t-tests. Error bars represent SEM.

Supplementary Figure 6

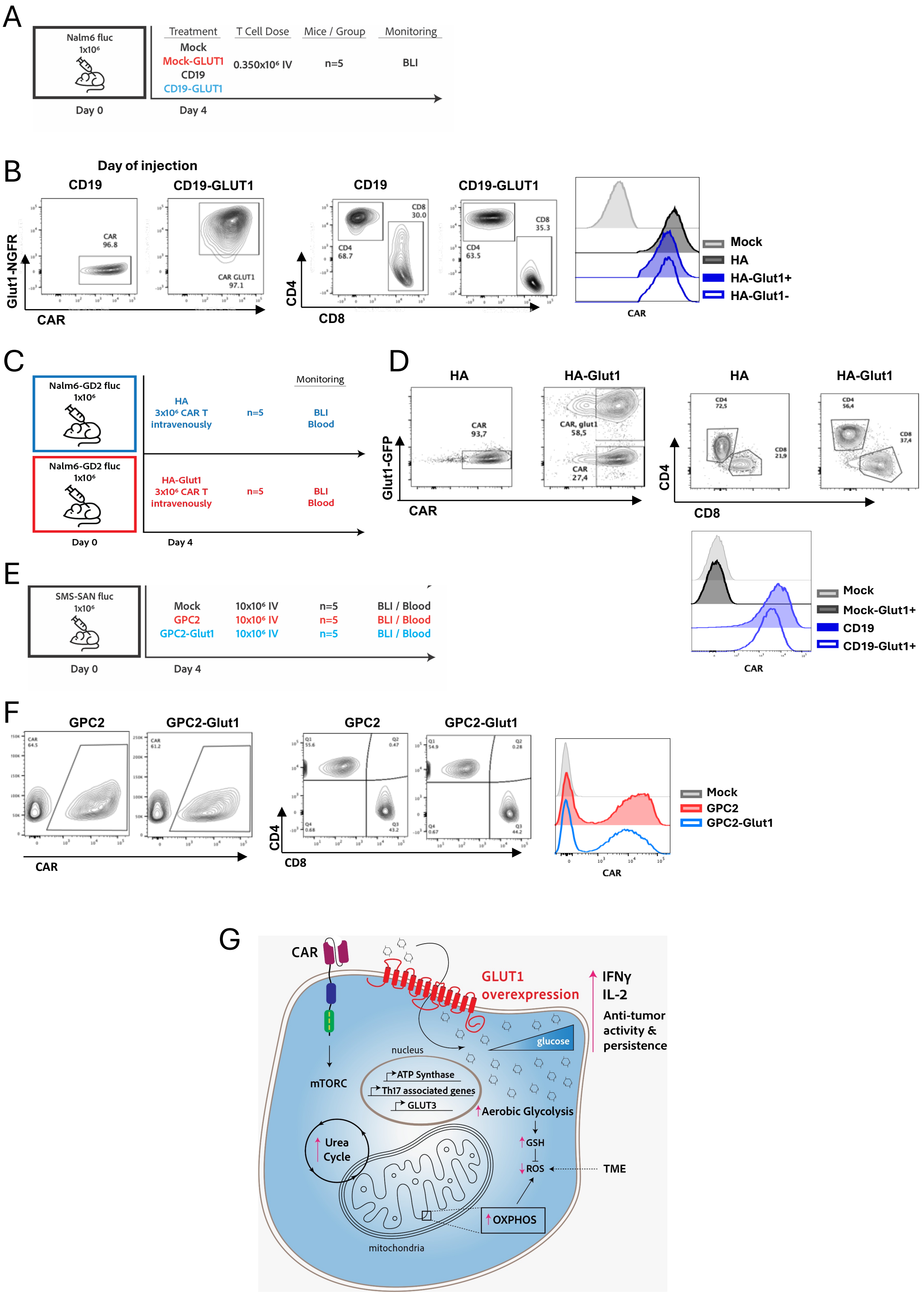

Supplemental Figure 6: Characterization of CAR-T ± GLUT1OE cells utilized for in vivo experiments, related to Figure 10

(A) Experimental design: NSG mice were injected intravenously (IV) with 1 × 10^6 Nalm6-fLuc tumor cells, and then 0.35 × 10^6 of CD19 ± GLUT1OE CAR-T cells IV on day 4. Tumor growth was monitored by BLI and spleens were harvested at endpoint for T cell and tumor quantifications.

(B) Characterization by flow cytometry of CD19 ± GLUT1OE CAR-T cells on day of *in vivo* injection.

(C) Experimental design: NSG mice were injected intravenously (IV) with 1 × 10^6 Nalm6-GD2+ fLuc tumor cells, and then 3 × 10^6 of HA ± GLUT1OE CAR-T cells IV on day 4. Tumor growth was monitored by BLI and blood was collected on days 25 and 40 for T cell quantification.

(D) Characterization by flow cytometry of HA ± GLUT1OE CAR-T cells on day of *in vivo* injection.

(E) Experimental design: NSG mice were injected intravenously (IV) with 1 × 10^6 SMS-SAN tumor cells, and then 10 × 10^6 of GPC2 ± GLUT1OE CAR-T cells IV on day 4. Tumor growth was monitored by BLI and blood was collected on days 18 and 34 for T cell quantification.

(F) Characterization of GPC2 ± GLUT1OE CAR-T cells on day of *in vivo* injection.

(G) GLUT1 overexpression induces metabolic changes and T cell differentiation. Upon activation, CAR-T cells increase cytokine secretion while suppressing ROS accumulation via GSH formation. This results in enhanced anti-tumor potency and persistence.
