# Supplementary material for: GLUT1 overexpression in CAR-T cells induces metabolic reprogramming and enhances potency
Source: Nat Commun. 2024 Oct 6;15:8658. doi: 10.1038/s41467-024-52666-y (PMC11456602; doi:10.1038/s41467-024-52666-y)
Supplement: Supplementary file 3 — Reporting Summary [file 41467_2024_52666_MOESM3_ESM.pdf]

nature portfolio

Corresponding author(s): Corresponding authors
Elena Sotillo: esotillo@stanford.edu
Crystal L. Mackall: cmackall@stanford.edu

Last updated by author(s): 8/14/2024

Reporting Summary

Nature Portfolio wishes to improve the reproducibility of the work that we publish. This form provides structure for consistency and transparency in reporting. For further information on Nature Portfolio policies, see our Editorial Policies and the Editorial Policy Checklist.

Statistics

For all statistical analyses, confirm that the following items are present in the figure legend, table legend, main text, or Methods section.

n/a	Confirmed	
☐	☒	The exact sample size (n) for each experimental group/condition, given as a discrete number and unit of measurement
☐	☒	A statement on whether measurements were taken from distinct samples or whether the same sample was measured repeatedly
☐	☒	The statistical test(s) used AND whether they are one- or two-sided<br>*Only common tests should be described solely by name; describe more complex techniques in the Methods section.*
☒	☐	A description of all covariates tested
☐	☒	A description of any assumptions or corrections, such as tests of normality and adjustment for multiple comparisons
☐	☒	A full description of the statistical parameters including central tendency (e.g. means) or other basic estimates (e.g. regression coefficient) AND variation (e.g. standard deviation) or associated estimates of uncertainty (e.g. confidence intervals)
☐	☒	For null hypothesis testing, the test statistic (e.g. F, t, r) with confidence intervals, effect sizes, degrees of freedom and P value noted<br>*Give P values as exact values whenever suitable.*
☒	☐	For Bayesian analysis, information on the choice of priors and Markov chain Monte Carlo settings
☒	☐	For hierarchical and complex designs, identification of the appropriate level for tests and full reporting of outcomes
☒	☐	Estimates of effect sizes (e.g. Cohen's d, Pearson's r), indicating how they were calculated

Our web collection on statistics for biologists contains articles on many of the points above.

Software and code

Policy information about availability of computer code

Data collection	FACSDiva (BD Biosciences) for flow cytometry data acquisition. LivingImage (Perkin Elmer) for IVIS Spectrum bioluminescent imaging acquisition. Incucyte ZOOM ver2016B (Essen BioScience) for incucyte image acquisition. Cytof software v7.1 (Helios) for mass cytometry.
Data analysis	FlowJo v10.9 (and older) (BD) for analysis of flow cytometry. STAR aligner, Gencode v36, DESeq2,DAVID (packages) for RNA seq analysis. GraphPad Prism v9 (and older) (GraphPad) for biostatistical analysis and graphing. Excel v16.77.1 (and older) (Microsoft) for spreadsheet management. SnapGene (GSL Biotech LLC) for vector visualization and cloning. ImageJ v1.51J (NIH) for image analysis. LivingImage ver4.5 (Perkin Elmer) for IVIS Spectrum bioluminescent imaging analysis. Incucyte ZOOM ver2016B (Essen BioScience) for incucyte image analysis and quantification. OMIQ for mass cytometry. MetaboAnalyst V5 for MS metabolite enrichment analysis. Progenesis QI software (v2.3) (Nonlinear Dynamics, Durham, NC) for Mass Spec.

For manuscripts utilizing custom algorithms or software that are central to the research but not yet described in published literature, software must be made available to editors and reviewers. We strongly encourage code deposition in a community repository (e.g. GitHub). See the Nature Portfolio guidelines for submitting code & software for further information.

Data

Policy information about availability of data

All manuscripts must include a data availability statement. This statement should provide the following information, where applicable:

- Accession codes, unique identifiers, or web links for publicly available datasets
- A description of any restrictions on data availability
- For clinical datasets or third party data, please ensure that the statement adheres to our policy

The main data supporting the findings of this study are available within the article and its Supplementary information. RNAseq data will be deposited into the Gene Expression Omnibus (GEO) repository upon publication. All other raw data generated during the study will be available from the corresponding authors upon reasonable request.

Research involving human participants, their data, or biological material

Policy information about studies with human participants or human data. See also policy information about sex, gender (identity/presentation), and sexual orientation and race, ethnicity and racism.

Reporting on sex and gender	Human leukocytes were obtained from Stanford Blood center and/or STEMCELL technologies and received based on availability and age range requested (<45 years of age). Sex was not a factor in choosing which samples were used in any studies of primary human blood content.
Reporting on race, ethnicity, or other socially relevant groupings	Race and ethnicity were not factors in choosing which samples were used in any studies of primary human blood content.
Population characteristics	For leukocytes obtained through Stanford Blood Center (SBC), population characteristics are reflective of healthy volunteers (as defined by SBC) of the Northern California Bay Area.
Recruitment	No authors in this study participated in recruitment of volunteers.
Ethics oversight	Stanford Internal Review Board protocol number 31287.

Note that full information on the approval of the study protocol must also be provided in the manuscript.

Field-specific reporting

Please select the one below that is the best fit for your research. If you are not sure, read the appropriate sections before making your selection.

☒ Life sciences ☐ Behavioural & social sciences ☐ Ecological, evolutionary & environmental sciences

For a reference copy of the document with all sections, see nature.com/documents/nr-reporting-summary-flat.pdf

Life sciences study design

All studies must disclose on these points even when the disclosure is negative.

Sample size	Our established animal models consistently yield low variance, and difference in the means between groups greater than 50%. Power calculation estimates determined that 5 animals per group was sufficient to determine statistical significance.
Data exclusions	No data were excluded from analysis
Replication	In vivo experiments were replicated at least 2 times with independent donors and experiments (as stated in the figure legends) with similar results.<br>In vitro experiments were typically run with triplicate technical replicates and were reproduced at least 2 times in independent experiments (as stated in the figure legends) with similar results.
Randomization	For in vivo tumor models, mice were randomized to ensure equal mean tumor burden before T cell transfer.
Blinding	Investigators were blinded during in vivo imaging acquisition. Otherwise, fully informed data analysis was performed.

Reporting for specific materials, systems and methods

We require information from authors about some types of materials, experimental systems and methods used in many studies. Here, indicate whether each material, system or method listed is relevant to your study. If you are not sure if a list item applies to your research, read the appropriate section before selecting a response.

Materials & experimental systems

n/a	Involved in the study	
☐	☒	Antibodies
☐	☒	Eukaryotic cell lines
☒	☐	Palaeontology and archaeology
☐	☒	Animals and other organisms
☒	☐	Clinical data
☒	☐	Dual use research of concern
☒	☐	Plants

Methods

n/a	Involved in the study	
☒	☐	ChIP-seq
☐	☒	Flow cytometry
☒	☐	MRI-based neuroimaging

Antibodies

Antibodies used	From METAFORA: GLUT1-specific H2RBD-GFP ligand (anti-GLUT1 binding ligand) (0.5 uL)<br>From BioLegend: CD4-APC-Cy7 (clone OKT4), CD8-PerCp-Cy5.5 (clone SK1), TIM-3-BV510 (clone F38-2E2), CD39-FITC, PE or APC-Cy7 (clone A1), IL-2-PE/Cy7 (clone MQ1-17H12), CD62L-BV605 (clone DREG-56), CD45RO-PE/Cy7 (clone UCHL1).<br>From eBioscience: PD-1-PE-Cy7 (clone eBio J105), LAG-3-PE (clone 3DS223H), CD45RO-PE-Cy7 (clone UCHL1), CD45-PerCp-Cy5.5 (clone HI30)<br>From BD: LAG-3-BV421 (clone T47-530), CD62L-BV605 (clone DREG-56), CD4-BUV395 (clone SK3), CD8-BUV805 (clone SK1), BrdU-PerCP-Cy5.5 (clone 3D4), CD271-BUV737 (clone C40-1457), Fixable Viability Stain 510 (0.4uL).<br>From Thermo Fisher Scientific: Phospho-S6-PE/Cy7 (clone cupk43k). Unless otherwise mentioned all antibodies were used at 2 uL per test.
Validation	All antibodies were validated by the manufacturer for reactivity to human antigens. All antibodies were either validated or recommended for flow cytometry.

Eukaryotic cell lines

Policy information about cell lines and Sex and Gender in Research

Cell line source(s)	Nalm6-GL was originally provided by Steve Grupp (CHOP). The 143b line was provided by C. Khanna (NCI, NIH) and were retrovirally transduced with GFP and luciferase (143B-GL), 293GP line by the Surgery Branch (NCI), SMS-SAN and NGP-GPC2 were generated at Children's Hospital of Philadelphia by Chris Bosse.
Authentication	STR DNA profiling of all cell lines is conducted by Genetica Cell Line testing once per year.
Mycoplasma contamination	Cell lines were tested once every six months for general use. Before using for in vivo experiments, cell lines are tested with MycoAlert detection kit (Lonza).
Commonly misidentified lines<br>(See ICLAC register)	*Name any commonly misidentified cell lines used in the study and provide a rationale for their use.*

Animals and other research organisms

Policy information about studies involving animals; ARRIVE guidelines recommended for reporting animal research, and Sex and Gender in Research

Laboratory animals	6-8 week old male or female NOD/SCID/IL2Rg (NSG) mice were used for all in vivo experiments.
Wild animals	The study did not involve wild animals.
Reporting on sex	Sex of animals was not considered in study design. All animals were sex and age matched in each experiment. The authors conclude that the findings are applicable, relevant, and reproducible for both males and females based on observed findings.
Field-collected samples	No field collected samples were used in this study.
Ethics oversight	Stanford Internal Review Board protocol number 31287.

Note that full information on the approval of the study protocol must also be provided in the manuscript.

Flow Cytometry

Plots

Confirm that:

☐ The axis labels state the marker and fluorochrome used (e.g. CD4-FITC).

☒ The axis scales are clearly visible. Include numbers along axes only for bottom left plot of group (a 'group' is an analysis of identical markers).

☐ All plots are contour plots with outliers or pseudocolor plots.

☐ A numerical value for number of cells or percentage (with statistics) is provided.

Methodology

Sample preparation	Up to 1 million T cells from culture were washed with D-PBS + 2% BSA (FACS Buffer), labeled in 100uL (or 50uL for 96 well format) FACS Buffer containing the relevant antibodies, and incubated at 4C in the dark for 20 minutes. Samples were washed 2X in 1mL FACS Buffer before running.
Instrument	BD LSRFortessa X-20
Software	FACSDiva for collection and FlowJo for analysis.
Cell population abundance	CD3+ T cells were enriched from leukocytes via negative selection prior to experimentation and analysis.
Gating strategy	All samples are gated on FSC/SSC lymphocyte populations, single cells (Using FSC-W/FSC-H and SSC-W/SSC-H), live populations, then CD4+ & CD8+. Frequency of positive gates is determined using an isotype or FMO control.

☒ Tick this box to confirm that a figure exemplifying the gating strategy is provided in the Supplementary Information.
